# Profile and Management of Traumatic Orbital Hematoma in a Child With Hemophilia C: A Case Report

**DOI:** 10.7759/cureus.97475

**Published:** 2025-11-21

**Authors:** Wejdan Al Mustafa, Saif K Aldossari, Muhammad A Afridi, Walaa Al-Dairi

**Affiliations:** 1 Department of Ophthalmology, Aljabr Eye and ENT Hospital, Al-Ahsa, SAU; 2 Department of Ophthalmology, King Faisal University, Al-Ahsa, SAU

**Keywords:** acute orbital compartment syndrome (aocs), childhood blindness, hemophilia, orbital decompression, orbital hematoma

## Abstract

Acute orbital compartment syndrome (AOCS) is a rare condition needing prompt surgical intervention to avoid irreversible visual loss. We present a two-year-old with decreased activity, head edema, pain, restricted ocular movement, proptosis, and downward displacement of the left eyeball after a minor fall. Upon thorough evaluation, hemophilia C was diagnosed. An MRI scan revealed a circumferential scalp hematoma and subperiosteal hematoma, causing proptosis of the left orbit. Surgical evacuation of the blood collection resulted in complete recovery and resolution of symptoms. In AOCS, bleeding diathesis must be ruled out, and prompt decompression treatment is recommended.

## Introduction

Acute orbital compartment syndrome (AOCS) is a rare surgical ophthalmic emergency in which a sudden rise in intra-orbital pressure compresses orbital structures, primarily the optic nerve and arterial blood supply. A rapid increase in fluid or mass in the orbit, mainly in the retrobulbar space, compresses the eyeball, its blood supply, and connections to the brain [1]. Irreversible visual loss may occur if prompt visual-saving procedures are delayed. Orbital hematoma is one cause of AOCS, in which blood accumulates in the retrobulbar muscle cone and originates from a ruptured short ciliary artery or subperiosteal vessels [2]. The condition needs to be differentiated from thyroid orbitopathy, blood collection following fracture of the orbital wall, and malignant tumors [3-5]. The ultrasound of the orbit and radioimaging are helpful in the diagnosis of a hematoma in the orbit [6]. Acute bilateral proptosis with orbital hematoma following trivial head trauma is reported at the emergency units of hospitals in older children with hemophilia as the underlying cause of hemorrhage [7,8]. However, to the best of our knowledge, children less than four years of age with hemophilia having a unilateral orbital hematoma are rare.

Hemorrhage in people with blood disorders is common, and even a minor trauma can trigger it. Hemophilia C is a rare blood disorder with a prevalence of one in one million of the population. Factor XI is low or absent in the blood in this disease, which leads to delayed clotting. It is also known as Rosenthal syndrome [9]. Minor head trauma in a child results in extensive subdural and intracranial bleeding that is asymptomatic and difficult to diagnose and usually affects both orbits.

We present a case of unilateral proptosis secondary to head trauma in a child with a family history of bleeding disorder in the mother. Its presentation, management, and outcomes are described.

## Case presentation

A two-year-old female child was brought to the ophthalmology emergency department with decreased activity and feeding, increased head size, and downward displacement of the left eyeball. She had minor head trauma due to a fall two days ago. On inquiry, parents reported that there was no history of vomiting or loss of consciousness after trauma. A history of bleeding disorders was reported from the mother's family. There was a consanguineous marriage between the child’s parents.

The team, comprising a pediatrician, a hematologist, a neurosurgeon, a general ophthalmologist, an oculoplastic surgeon, and an anterior segment consultant, evaluated the child at the request of the emergency physician. The child was irritable and anxious but conscious and aware of her surroundings. The blood pressure was 105/76 mmHg, the temperature was 36.7 °C, the pulse rate was 95 bpm, the respiratory rate was 25 per minute, and the oxygen saturation was 98% on room air. The pediatric examination of cardiovascular, respiratory, gastrointestinal, genitourinary, musculoskeletal, and neurological systems was unremarkable.

On examination, we noted severe generalized head edema, restricted and painful ocular movements, proptosis, and downward displacement of the left eyeball. The child could see hand movement using the left eye, but the vision was more than six meters in the right eye. Eye movement testing was difficult because the child was crying while moving their left eye in different directions. The venous blood was withdrawn and sent for laboratory evaluation (Table 1).

**Table 1:** Initial laboratory results of the child with unilateral orbital hematoma with hemophilia.

Blood Tests		Result	Reference Range
Complete Blood Count (CBC)	White Blood Cell (WBC)	12.16 10^3^/uL	(6- 17 10^3^/uL)
	Red Blood Cell (RBC)	2.56 10^6^/uL	(3.9- 5.0 10^6^/uL)
	Hemoglobin (HGB)	6.3 g/dL	(11.0- 13.8 g/dL)
	Mean Corpuscular Volume (MCV)	80.9 fL	(76- 90 fL)
	Platelet (PLT)	166 10^3^/uL	(150- 350 10^3^/uL)
	Hematocrit (HCT)	20.70%	(32- 40%)
Biochemistry	Urea	2.0 mmol/L	(1.7- 8.3 mmol/L)
	Creatinine	26 umol/L	(53- 115 umol/L)
	Aspartate Aminotransferase (AST)	162.3 U/L	(15- 37 U/L)
	Alanine Aminotransferase (ALT)	14.8 U/L	(0- 35 U/L)
	Lactate Dehydrogenase (LDH)	408 U/L	(100- 190 U/L)
	Serum Sodium (Na^+^)	103.0 mmol/L	(98- 115 mmol/L)
	Serum Potassium (K^+^)	5.06 mmol/L	(3.4- 5.2 mmol/L)
Coagulation Profile	Prothrombin Time (PT)	15.3 sec	(11- 15 sec)
	Partial Thromboplastin Time (PTT)	59.8 sec	(26- 40 sec)
	International Normalization Ratio (INR)	1.12	(0.8- 1.1)
Coagulation Factors	Factor II	71.50%	(70- 120%)
	Factor V	83%	(94-1 41%)
	Factor IX	89%	(64- 216%)
	Factor X	88%	(53- 122%)
	Factor XI	13%	(62- 125%)
	von Willebrand Factor (VWF) Antigen	96.10%	(50- 160%)
	von Willebrand factor VWF Assay	23.70%	(47.8- 173.2%)
HB Electrophoresis	Hemoglobin Hb A	97.90%	(96.8- 97.8%)
	Hemoglobin HB A2	2.10%	(2.2- 3.2%)

The child was admitted, and urgent multiple-packed RBC and fresh frozen plasma transfusions were carried out in the pediatric intensive care unit under the supervision of a pediatrician and a hematologist. In addition, tranexamic acid, broad-spectrum antibiotics, and systemic steroids were administered intravenously.

**Figure 1:**
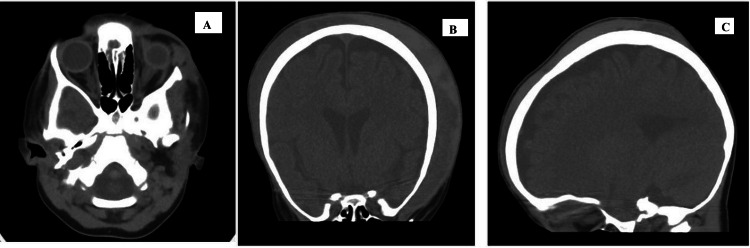
Computed tomography scan of skull and orbits of the child with left orbital hematoma and scalp hematoma. A circumferential scalp hematoma crossing the sutures with evidence of mixed acute and subacute blood density on the left side of the scalp. A left superior orbital roof subperiosteal collection measuring 2.0x0.7x0.7 cm caused proptosis of the left orbit. There was no subarachnoid, intracerebral, or intraventricular hemorrhage.

Radioimaging investigations included a computed tomography (CT) scan of the brain and orbit. The radiologist confirmed a circumferential scalp hematoma crossing the sutures with evidence of mixed acute and subacute blood density on the left side of the scalp. A left superior orbital roof subperiosteal collection measuring 2.0x0.7x0.7 cm caused proptosis of the left orbit. No subarachnoid, intracerebral, or intraventricular hemorrhage was noted (Figure 1).

**Figure 2:**
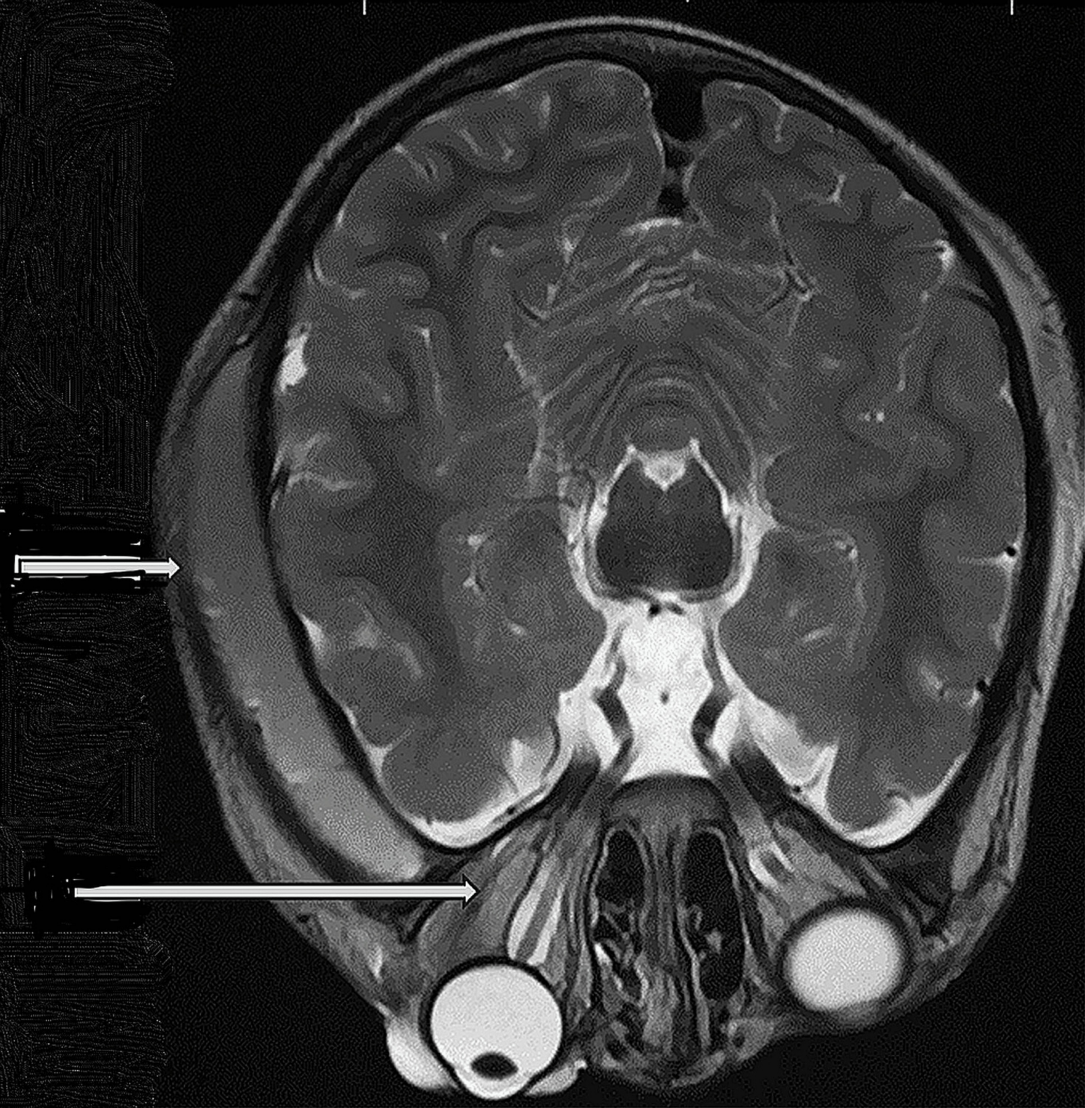
Magnetic resonance tomography of the skull and orbit of the child with left orbital hematoma and scalp hematoma. The image shows extensive circumferential scalp swelling and crossing suture lines, most prominent in the left parietal region. Post-septal extraconal intraorbital hyperacute hemorrhage (30.5 x 24 mm) pushing the globe forward and downward, and eyelid edema. The arrow shows the presence of intra-orbital and subperiosteal blood collection.

Magnetic resonance imaging of the brain showed extensive circumferential scalp swelling, crossing suture lines, most prominent in the left parietal region. Susceptibility-weighted imaging showed a likely acute/subacute subgaleal hemorrhage with underlying edema. Left severe proptosis secondary to mixed signal post-septal extraconal intraorbital hyperacute hemorrhage, measuring about 30.5 x 24 mm, pushing the globe forwardly and downwardly, associated with eyelid edema (Figure 2).

After the child was given general anesthesia, we conducted an ophthalmological examination of the anterior and posterior segments of the left eye. We noted a corneal epithelial defect (CED), diffuse chemosis, faint subconjunctival hemorrhage, sluggish pupillary reaction, supraorbital fullness, and normal dilated fundus examination.

The corneal epithelium of the left eye was debrided. The orbital hematoma was drained and evacuated through a 2-cm central sub-brow incision. Multiple cycles of saline irrigation and aspiration were performed to ensure total hematoma aspiration. A significant reduction of proptosis was noted intraoperatively. The drainage incision was sutured with 6/0 Vicryl, and a pressure patch was applied.

**Figure 3:**
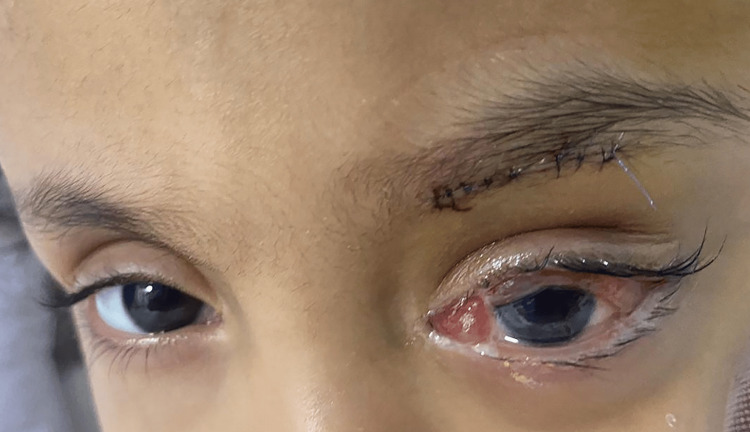
Status of the left orbit of the child with hemophilia C four weeks after treating the orbital hematoma with proptosis.

The child was hospitalized for 11 days. On postoperative day one, the CED healed with complete subsidence of the proptosis, subconjunctival hemorrhage, and chemosis (Figure 3).

The head circumference decreased from 54 cm to 52 cm at the initial presentation. On the day of discharge, the child demonstrated central, steady, and maintained CSM visual preference with total extraocular motility. His vision was more than six meters in both eyes. The child was brought for follow-up fortnightly. Both head circumference and orbital volume were normalized, and chemosis and lid edema in the left eye resolved. The pupillary reaction in both eyes was equal and brisk. 

The parents were counseled by a genetic counselor, who explained the prognosis and care to be taken for a child and newer treatment modalities available for hemophilia.

**Figure 4:**
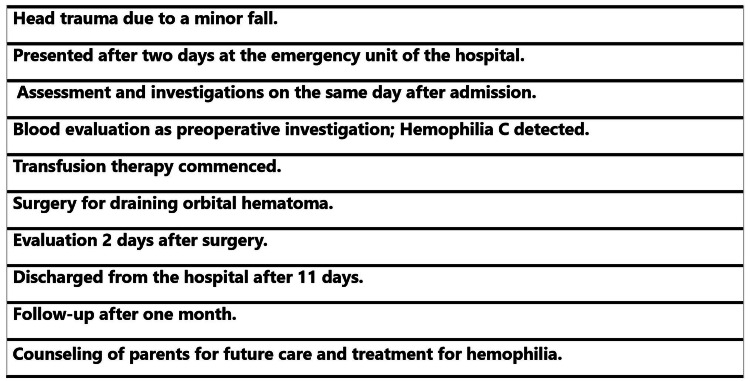
Timeline of events for presentation and management of orbital hematoma with proptosis in the child with hemophilia.

The timeline of assessment, intervention, and follow-up is given in Figure 4.

## Discussion

This case of orbital hematoma following minor head trauma is perhaps the first in Saudi Arabia and the Middle East where rare bleeding diathesis was detected on comprehensive assessment. Prompt detection and management could restore orbital status and preserve vision in the left eye. Although the radioimaging investigation showed that the scalp hematoma crossed the sutures, the unilateral hematoma in the left eye remains unexplained. Orbital bone damage in such cases should be explored in detail before concluding that the scalp hematoma has spread to the left orbit.

The orbital hematoma is an emergency and demands prompt intervention to preserve vision. Despite its relatively infrequent occurrence compared to other orbital pathologies, orbital hematoma, especially in persons with bleeding disorders, demands accurate diagnosis and timely intervention to mitigate adverse outcomes. This condition could arise from different aetiologies, such as trauma, vascular abnormalities, surgical intervention, and coagulation disorders, leading to a spectrum of clinical characteristics ranging from pain, diplopia, subconjunctival hemorrhage, periorbital ecchymosis, reduced visual acuity, exophthalmos, ophthalmoplegia, optic disc swelling, and afferent pupillary defect [1,10].

Imaging modalities provide valuable insight into etiology. Computed tomography (CT) and magnetic resonance Imaging (MRI) enable the caregiver to detect pathology at early stages, localize the underlying cause of orbital hematomas, provide prognostic information, and monitor the impact of therapeutic interventions [11]. The management algorithm includes conservative approaches such as analgesia, antiemetics, and cold compression for minor hematomas. At the same time, surgical decompression, in the form of lateral canthotomy and inferior cantholysis, is reserved for vision-threatening hematomas that could lead to irreversible ischemia. More aggressive surgical measures, specifically hematoma evacuation and surgical orbital decompression, can be planned if these measures fail to improve visual acuity [12].

Hemophilia is an X-linked recessive bleeding disorder. The two main subtypes are Hemophilia A and Hemophilia B. The inheritance pattern for hemophilia C is autosomal dominant or recessive, depending on the mutation in the F11 gene on chromosome 4. Reduced level or activity of factor XI, also known as hemophilia C (Rosenthal syndrome) or acquired hemophilia, which results in moderate bleeding symptoms, usually occurring after trauma or surgery, has a prevalence of 1 per million population [12,13]. Concurrent management of hemophilia is crucial to make surgical procedures safe and prevent recurrent hemorrhage. The guidelines for managing persons with hemophilia are described in detail [14,15]. The health staff of the emergency unit, child health care, and ophthalmologists should be aware of the evolution and availability of treatment, the need for teamwork, and the agencies that support the needs of hemophiliacs to counsel the parents of children with hemophilia [16].

Family history of bleeding disorder and consanguineous marriage among parents helped to suspect bleeding disorder as the underlying cause in this case. However, one should rule out the possibility of child abuse, especially in a girl child.

Comprehensive management of a child with hemophilia C is lifelong and involves teamwork. Deficiencies noted in the blood test should be discussed with a hematologist, pathologist, neurologist, and pediatrician to determine the need for therapy beyond ophthalmic manifestations. The anemia noted in the present study seems to be due to the ignorance of caregivers and parents, even if the mother had a history of blood disorder in the family, and there was a history of consanguineous marriage.

## Conclusions

A case of orbital hematoma in a young child at the emergency clinic of a hospital is rare and a challenge for caregivers and needs teamwork for comprehensive assessment and prompt management to avoid life-threatening sequelae, as well as address the underlying cause of hemophilia C in this child. A thorough evaluation, including a history of hematoma on trivial injuries, genetic blood disorder, laboratory tests, ultrasonography, and radioimaging of the brain and orbit, helps to diagnose the condition. Depending on the sight-threatening compression of the optic nerve and retinal changes, one should plan for emergency decompression rather than allowing the hematoma to regress naturally.

## References

[1] Murali S, Davis C, McCrea MJ, Plewa MC (2021). Orbital compartment syndrome: pearls and pitfalls for the emergency physician. J Am Coll Emerg Physicians Open.

[2] McCallum E, Keren S, Lapira M, Norris JH (2019). Orbital compartment syndrome: an update with review of the literature. Clin Ophthalmol.

[3] Nowak M, Nowak W, Marek B, Kos-Kudła B, Siemińska L, Londzin-Olesik M, Kajdaniuk D (2024). Differential diagnosis of thyroid orbitopathy - diseases mimicking the presentation or activity of thyroid orbitopathy. Endokrynol Pol.

[4] Narjus-Sterba M, Puolakkainen T, Kokko L, Thorén H, Snäll J (2025). Occurrence and outcomes of retrobulbar haematoma in 2149 orbital fracture patients. Oral Maxillofac Surg.

[5] Vogele D, Sollmann N, Beck A (2022). Orbital tumors—clinical, radiologic and histopathologic correlation. Diagnostics (Basel).

[6] Lanni V, Iuliano A, Fossataro F (2021). The role of ultrasonography in differential diagnosis of orbital lesions. J Ultrasound.

[7] Kim SY, Cha HG, Jang SY, Hwang SC (2021). Delayed massive expansion of subgaleal hematoma complicated with proptosis in hemophilia B. Korean J Neurotrauma.

[8] Pujari A, Mukhija R, Shashni A, Obedulla H, Meel R, Bajaj MS (2018). Bilateral hemorrhagic proptosis due to an uncommon cause in ocular emergency. Indian J Ophthalmol.

[9] Kianian S, Scorsese G, Zabirowicz E, Poppers J (2023). Perioperative management of a patient with hemophilia C and allergy to fresh frozen plasma. Case Rep Anesthesiol.

[10] Tawfik HA, Fouad YA, Hamza YA (2017). Retrobulbar hemorrhage: etiology, pathogenesis, epidemiology, and clinical perspectives. Emergencies of the Orbit and Adnexa.

[11] Gavrel M, Rafowicz A, d'Oiron R, Franchi-Abella S, Lambert T, Adamsbaum C (2019). Imaging features of atypical bleeds in young patients with hemophilia. Diagn Interv Imaging.

[12] Soare S, Foletti JM, Gallucci A, Collet C, Guyot L, Chossegros C (2015). Update on orbital decompression as emergency treatment of traumatic blindness. J Craniomaxillofac Surg.

[13] Alblaihed L, Dubbs SB, Koyfman A, Long B (2022). High risk and low prevalence diseases: hemophilia emergencies. Am J Emerg Med.

[14] Razaq M, Chouhan M (2020). Clinical profile of hemophilia in children in a tertiary care hospital in North India. JK Science.

[15] Srivastava A, Santagostino E, Dougall A (2020). WFH guidelines for the management of hemophilia, 3rd edition. Haemophilia.

[16] Marchesini E, Morfini M, Valentino L (2021). Recent advances in the treatment of hemophilia: a review. Biologics.

